# Molecular Expression and Pharmacological Evidence for a Functional Role of Kv7 Channel Subtypes in Guinea Pig Urinary Bladder Smooth Muscle

**DOI:** 10.1371/journal.pone.0075875

**Published:** 2013-09-20

**Authors:** Serge A. Y. Afeli, John Malysz, Georgi V. Petkov

**Affiliations:** Department of Drug Discovery and Biomedical Sciences, South Carolina College of Pharmacy, University of South Carolina, Columbia, South Carolina, United States of America; University of Texas Health Science Center, United States of America

## Abstract

Voltage-gated Kv7 (KCNQ) channels are emerging as essential regulators of smooth muscle excitability and contractility. However, their physiological role in detrusor smooth muscle (DSM) remains to be elucidated. Here, we explored the molecular expression and function of Kv7 channel subtypes in guinea pig DSM by RT-PCR, qRT-PCR, immunohistochemistry, electrophysiology, and isometric tension recordings. In whole DSM tissue, mRNAs for all Kv7 channel subtypes were detected in a rank order: Kv7.1~Kv7.2Kv7.3~Kv7.5Kv7.4. In contrast, freshly-isolated DSM cells showed mRNA expression of: Kv7.1~Kv7.2Kv7.5Kv7.3~Kv7.4. Immunohistochemical confocal microscopy analyses of DSM, conducted by using co-labeling of Kv7 channel subtype-specific antibodies and α-smooth muscle actin, detected protein expression for all Kv7 channel subtypes, except for the Kv7.4, in DSM cells. L-364373 (R-L3), a Kv7.1 channel activator, and retigabine, a Kv7.2-7.5 channel activator, inhibited spontaneous phasic contractions and the 10-Hz electrical field stimulation (EFS)-induced contractions of DSM isolated strips. Linopiridine and XE991, two pan-Kv7 (effective at Kv7.1-Kv7.5 subtypes) channel inhibitors, had opposite effects increasing DSM spontaneous phasic and 10 Hz EFS-induced contractions. EFS-induced DSM contractions generated by a wide range of stimulation frequencies were decreased by L-364373 (10 µM) or retigabine (10 µM), and increased by XE991 (10 µM). Retigabine (10 µM) induced hyperpolarization and inhibited spontaneous action potentials in freshly-isolated DSM cells. In summary, Kv7 channel subtypes are expressed at mRNA and protein levels in guinea pig DSM cells. Their pharmacological modulation can control DSM contractility and excitability; therefore, Kv7 channel subtypes provide potential novel therapeutic targets for urinary bladder dysfunction.

## Introduction

The physiological roles of voltage-gated K^+^ (Kv) channels are now emerging [1,2,3,4,5,6,7]. Evidence suggests that they might be critical regulators of detrusor smooth muscle (DSM) excitability and contractility [1,8]. In general, the opening of K^+^ channels causes membrane hyperpolarization, closure of L-type voltage-dependent Ca^2+^ channels, reduction of net Ca^2+^-influx, and smooth muscle relaxation. Inhibition of K^+^ channels has an opposite effect promoting contractility [1,8]. Among the 40 genes encoding Kv channels identified in the human genome, the functions of the five pore-forming α subunits of the Kv7 subfamily are not well understood in DSM [9]. Specifically, uncertainties remain for Kv7.1, Kv7.2, Kv7.3, Kv7.4, and Kv7.5 channel subtypes encoded by KCNQ1, KCNQ2, KCNQ3, KCNQ4, and KCNQ5 genes, respectively [10]. The Kv7 channel α subunits share a common homology. Similar to other Kv channels, they are structurally composed of six transmembrane domains (TM1-TM6) with cytoplasmic N- and C-termini and a single pore loop between TM5-TM6 and a voltage sensor within TM4 [10]. The Kv7 channel α subunits assemble as tetramers of homo- or hetero-tetrameric subunits with preferential combinations such as: Kv7.2/Kv7.3 and Kv7.3/Kv7.5 complexes [11]. In addition, Kv7 channels may associate with regulatory β subunits encoded by KCNE genes (KCNE1-5) that fine-tune the functional and biophysical properties, and influence the plasma membrane expression of the Kv7 channel assemblies [12].

Kv7 channel subtypes have distinct properties and tissue distribution profiles [11,13,14]. As measured in recombinant based systems, the half-maximum activation constants for the different Kv7 channels range from -60 to -20 mV. The effects of Ca^2+^/calmodulin and cAMP signaling pathways and pharmacological sensitivities to compounds also depend on the α/β subunit composition [11,13,14]. In rodent and human blood vessels, evidence points to the predominant roles of Kv7.1 and Kv7.4 channel subtypes regulating vascular smooth muscle membrane potential and contractility [5,15]. Kv7 channels, in particular Kv7.4 and Kv7.5 channel subtypes, are present throughout the gastrointestinal tract and are proposed to influence spontaneous and electrical field stimulation (EFS)-evoked contractions [16,17]. In airway smooth muscle, Kv7.1, Kv7.2, Kv7.4, or Kv7.5 channel subtypes demonstrate species-specific expressions and likely regulate the airway diameter and, thus, bronchoconstriction [2]. In the heart, the expressed Kv7.1 channels co-assembled with KCNE1 peptides underlie the voltage-dependent delayed rectifier that contributes to the late repolarization phase of the cardiac action potential [13,18]. Neuronal heteromeric Kv7 channels incorporating Kv7.2, Kv7.3, Kv7.4 or Kv7.5 channel subtypes are expressed in various brain regions, and they control the membrane potential and action potential pattern generation [19]. Pharmacological potentiation of heteromeric Kv7.2/Kv7.3 channels is thought to underlie the primary mechanism of action for retigabine, a recently approved anti-epileptic drug [20].

Physiological roles of Kv7 channel subtypes in the urinary bladder remain to be fully elucidated. When investigating the effects of retigabine in clinical trials for epilepsy, patients taking the drug showed higher incidence of urinary retention than those on placebo [21]. This finding and sporadic few other studies suggest that Kv7 channels might be important regulators of DSM function. Specifically, acute retigabine exposure has been shown to increase micturition volume and voiding intervals in rats [22,23], reduce the contractility and tone of rat and pig DSM isolated strips [23,24,25], and enhance Kv7 currents in guinea pig urinary bladder interstitial cells [26]. In addition, chronic exposure to retigabine resulted in a higher incidence of distended urinary bladders in a pre-clinical study [21]. Kv7 channel subtype expression has been previously studied in rats by qRT-PCR, demonstrating the highest mRNA expression for Kv7.4 channels followed by detectable levels of Kv7.5 and Kv7.1 and minimal expression of Kv7.2 and Kv7.3 channel subtypes in whole bladder tissues [23]. Another study, also in rats, failed to detect any transcripts for Kv7.1, Kv7.2, or Kv7.3 channel subtypes by RT-PCR in whole bladder tissues; however, positive expression of KCNE1 and KCNE2 mRNAs was found [27]. In comparison, Kv7.3-Kv7.5 mRNAs were detected with the apparent highest expression of Kv7.4 channel subtype by qRT-PCR in pig DSM [25]. While our article was under review, a study reported the expression of all Kv7.1-Kv7.5 channel subtypes in cells derived from whole guinea pig bladder (urothelium-free) and also provided evidence for their roles using patch-clamp electrophysiology and isometric tension recordings of DSM spontaneous phasic contractions [28]. Whether the expression of Kv7 channels in whole DSM tissue originated directly from DSM cells, interstitial cells, urothelial cells, nerve cells, or another cell type, remains to be established.

In the present study, we focused on DSM cells as they are key facilitators of urinary bladder function. We investigated the molecular expression (mRNA and protein) of all Kv7 channel subtypes (Kv7.1-7.5). We further used Kv7 channel activators and inhibitors to determine whether the pharmacological modulation of Kv7 channels affects DSM spontaneous and nerve-evoked contractions. To our knowledge, no previous study investigated the expression of Kv7 channel subtypes in guinea pig DSM cells with confocal microscopy detecting the co-labelling of Kv7 channel subtype and smooth muscle markers, single-cell qRT-PCR, and membrane potential recordings by the perforated whole cell patch-clamp technique in response to retigabine. We also determined the functional effects of Kv7 channel modulators using full-concentration responses on DSM contractility, both spontaneous phasic and EFS-induced contractions. As the guinea pig is a widely used experimental animal model to study the properties of DSM [1,29,30,31,32,33,34,35,36], our study addresses important questions relating to the Kv7 channel subtypes, elucidating their functional roles and potential utility as novel targets for urinary bladder disorders.

## Materials and Methods

### DSM tissue harvesting

Animal experiments were conducted in accordance with the Animal Use Protocol #1747 reviewed and approved by the University of South Carolina Institutional Animal Care and Use Committee. For this study, 49 adult male Hartley-Albino guinea pigs (Charles River Laboratories, Raleigh, NC, USA), weighing on average 421.5±9.2 g, were euthanized by carbon dioxide inhalation followed by thoracotomy. The whole bladder was then removed by transversal incision above the bladder neck.

### DSM cells isolation and collection

Guinea pig DSM cells were freshly-isolated and collected as previously described [32,34,35,36]. The bladder was sliced open then the urothelium and mucosa were removed under a dissection microscope using microscissors. The DSM was then cut into strips (5–8 mm long, 2–4 mm wide), and 1–2 strips were placed in dissection solution (2 ml) supplemented with 1 mg/ml bovine serum albumin (BSA; Fisher Scientific, Fair Lawn, NJ, USA), 1 mg/ml papain (Worthington Biomedical Corp., Lakewood, NJ, USA), 1 mg/ml DL-dithiothreitol (Sigma-Aldrich, St. Louis, MO, USA), and incubated for 12–30 min at 37 °C. Next, the DSM strips were transferred into dissection solution (2 ml) containing 1 mg/ml BSA, 0.5-1 mg/ml collagenase type II (Sigma-Aldrich or Worthington Biomedical Corp.), 1 mg/ml tripsin inhibitor (MB Biomedical LLC, Solon, OH, USA), and 100 µM CaCl_2_ and incubated for another 8-15 min at 37 °C. DSM strips were then washed with fresh BSA containing dissection solution. The individual DSM cells were dispersed when the enzyme-treated tissue was passed through the tip of a fire-polished Pasteur pipette. Dispersed DSM cells were put in a glass-bottom chamber and allowed to settle for about 20 min. Next, DSM cells were washed out 4-5 times using a perfusion system, which removes the cells that do not adhere to the glass-bottom of the chamber. DSM cells were characterized by their spindle-shape, bright and shiny appearance under the Axiovert 40CFL microscope (Carl Zeiss^®^, Oberkochen, Germany) as well as their responsiveness to mechanical stimulation that indicates the presence of functional contractile proteins and that they are physiologically active. DSM cells were used in electrophysiological experiments or collected individually by suction into a glass micropipette using an MP-285/ROE micromanipulator (Sutter Instruments, San Rafael, CA, USA). A pool of approximately 100 DSM cells was collected for RNA extraction and further single-cell RT-PCR and qRT-PCR experiments.

### RT-PCR and qRT-PCR experiments

Using the RNeasy Mini Kit (Qiagen, Hilden, Germany), we extracted total RNA from guinea pig whole DSM tissue, brain tissue, and a pool of enzymatically freshly-isolated DSM cells as previously described [35,37]. The extracted total RNA was reverse-transcribed into cDNA using M-MLV Reverse transcriptase (Promega, Madison, WI, USA) and oligo d(T) primers. The amplification of the cDNA product was performed in the presence of GoTaq Green Master Mix (Promega) and PCR primers for Kv7.1, Kv7.2, Kv7.3, Kv7.4, and Kv7.5 channels, respectively, using a mastercycler gradient from Eppendorf (Hamburg, Germany). We used previously published guinea pig primers sequences for Kv7.1–7.5 channels [38]. The primer sequences and the size of the amplicons were as follows: Kv7.1-F:5’-ATTGTCCTGGTGGTGTTCTTTG-3′ and R: 5′-CCCCTGATGGCTGATGTGG-3′ (206 bp); Kv7.2- F: 5′-TCTACGCCACCAACCTGTC-3′ and R: 5′-TACATGGGCACCGTGACC-3′ (79 bp); Kv7.3- F: 5'-CTTGAAAACCGTCATCTGC-3' and R: 5'-CAAGTTCACAGGGTCGTG-3' (124 bp); Kv7.4- F: 5'-CGATGTCATGCCTGCTGTG-3' and R: 5'-GGTGTCCTGCTGAATACTGC-3' (137 bp); Kv7.5- F: 5'-CGTCCGCACTCAGAAGTC-3' and R: 5'-TCCAATGTACCAGGCTGTG-3' (137 bp); GAPDH- F: 5′-TACGACAAGTCCCTCAAGATTG-3′ and R: 5′-TCTGGGTGGCAGTGATGG-3′ (139 bp) [38]. Kv7 channel primers were synthesized by Integrated DNA Technologies (IDT, Coralville, IA, USA). In the RT-PCR experiments, cDNA products from guinea pig whole DSM tissue, brain tissue, and DSM isolated cells were heated for 5 min at 95°C then amplified by 35 cycles (95°C for 30 s, 58°C for 30 s, 72°C for 30 s) followed by a 5min extension at 72°C. PCR products were then loaded onto a 2% agarose ethidium bromide-stained gel and allowed to migrate via electrophoresis. The expected length of the fragment for each Kv7 channel subtype was determined by using a 20 bp extended range DNA ladder from Lonza (Rockland, ME, USA). Guinea pig brain tissue was used as a positive control. In the qRT-PCR experiments, we applied a two-step amplification followed by a melting curve protocol using the IQ ^TM^5 Thermo Cycler system (Bio-Rad, California, USA). Ten ng/µL of cDNA from guinea pig whole DSM tissue or isolated DSM cells was mixed with IQ SYBR Green Supermix (Bio-Rad, California, USA). Each individual Kv7 channel subtype primer set was separately loaded onto a 96-well plate. GAPDH was chosen as an internal control gene to analyze Kv7 channel subtype mRNA relative expression; each sample was run in triplicate. The parameters of the qRT-PCR experiments were as follows: cycle 1, 95°C for 3 min; cycle 2, 95°C for 10 s then 58°C for 30 s (repeated 40 times). After amplification, melting curve analyses from 61°C to 101.5°C in 0.5 °C increments every 10 s were conducted to assure correct PCR product quality. All RT-PCR products from the whole DSM tissue, brain tissue, and DSM isolated cells were purified using the GenElute^™^ PCR Clean-Up Kit (Sigma-Aldrich Co) and sequenced at the University of South Carolina Environmental Genomics Core Facility (Columbia, SC, USA) to confirm their identity.

### Immunohistochemistry

Immunohistochemistry experiments were performed to determine whether Kv7 channel proteins were expressed in guinea pig DSM cells following the method described previously [35], with minor modifications. Guinea pig whole DSM tissue was sliced into thin sections (~100 µm) using a vibratome Model G tissue slicer (Oxford Laboratories, Foster City, CA, USA) and transferred to individual dishes for immunostaining. Tissue sections were incubated with one of the following primary antibodies: anti-KCNQ1 (Kv7.1) (1:100, Santa Cruz Biotechnology, Santa Cruz, CA, USA, cat. number: SC-10646), anti-KCNQ2 (Kv7.2) (1:100, Santa Cruz Biotechnology, cat. number: SC-7793), anti-KCNQ3 (Kv7.3) (1:100, Santa Cruz Biotechnology, cat. number: SC-7794), anti-KCNQ4 (Kv7.4) (1:100, Santa Cruz Biotechnology, cat. number: SC-20882), or anti-KCNQ5 (Kv7.5) (1:100, Santa Cruz Biotechnology, cat. number: SC-18048) in 1% BSA/PBS at 37°C for 1 h. DSM tissue sections were then rinsed multiple times with 1% BSA/PBS, incubated for one hour in 5% normal donkey serum in 1% BSA/PBS, then re-incubated with secondary antibody (1:100) [(Cy3-conjugated anti-goat IgG) (Jackson ImmunoResearch, West Grove, PA, USA)] in the dark for another hour. Tissue sections were subsequently washed with 1% BSA/PBS then PBS and then incubated with α-smooth muscle actin-FITC antibody (F3777, Sigma-Aldrich) in PBS for 2 h in the dark. Next, DSM tissue sections were rinsed three or more times with PBS, incubated with 4′,6-diamidino-2-phenylindole (DAPI) (1:5000) in PBS for 15 min and then mounted with 1,4-diazabicyclo[2.2.2] octane (DABCO) (Sigma-Aldrich). Tissue sections were visualized under a confocal microscope LSM 700 META (Carl-Zeiss^®^) with 63x oil immersion objective.

### Isometric DSM tension recordings

Isometric DSM tension recordings were performed following previously published procedures [32,34,35,36]. Guinea pig DSM strips were clipped between a force displacement transducer and a hook, then submerged into a thermostatically controlled (37°C) 10 ml bath filled with Ca^2+^-containing physiological saline solution (PSS) and gassed with 95% oxygen-5% carbon dioxide. DSM strips were then stretched to 1 g of tension to elicit spontaneous phasic contractions. Strips were washed out with fresh PSS every 15 min and allowed to equilibrate for a period of 45 min to 1 h. DSM isolated strips that exhibited spontaneous phasic contraction amplitude >0.1 g were treated with tetrodotoxin (TTX, 1 µM), a neuronal Na^+^ channel blocker, for at least 15 min before application of increasing concentrations of a Kv7 channel modulator. We activated Kv7 channels by applying increasing concentrations (100 nM -100 µM) of L-364373 (also known as R-L3), a Kv7.1 potentiator, or retigabine, a Kv7.2–7.5 channel activator, at 10-min intervals. We also evaluated DSM spontaneous phasic contractions responses to two pan-Kv7 (equally effective at Kv7.1 – Kv7.5 subtypes) channel blockers XE991 and linopiridine cumulatively added separately (100 nM -30 µM). In DSM strips, which exhibited spontaneous phasic contraction amplitudes <0.1 g, contractions were induced by electrical field stimulation (EFS) in the absence of TTX. Using a PHM-152I stimulator (Med Associates, Inc., Georgia, VT, USA), we either applied stimulation of 10 Hz EFS frequency at 1 min intervals or we applied increasing EFS frequencies (0.5, 2, 3.5, 5, 7.5, 10, 12.5, 15, 20, 30, 40, 50 Hz) at 3 min intervals. On the 10 Hz EFS-induced contractions, increasing concentrations of Kv7 channel activators or inhibitors were applied as described above. On the 0.5–50 Hz EFS-induced contractions, a single concentration of a Kv7 channel modulator was applied as indicated. The EFS pulses had the following parameters: pulse amplitude (20 V), pulse width (0.75 ms), stimulus duration (3 s), polarity was reversed for alternating pulses. The DSM responses to EFS was recorded using MyoMed software (Med Associates).

### Perforated whole cell patch-clamp recordings

We applied the amphotericin-B perforated whole cell patch-clamp technique to record the membrane potential in the current-clamp mode (I=0) as described previously [33,34,36].

### Solutions and drugs

The Ca^2+^-free dissection solution had the following composition (in mM): 80 monosodium glutamate; 55 NaCl; 6 KCl; 10 glucose; 10 N-2-hydroxyethylpiperazine-N'-2-ethanesulphonic acid (HEPES); 2 MgCl_2_; and pH 7.3 adjusted with NaOH. The Ca^2+^-containing PSS was prepared daily and contained (in mM): 119 NaCl; 4.7 KCl; 24 NaHCO_3_; 1.2 KH_2_PO_4_; 2.5 CaCl_2_; 1.2 MgSO_4_; 11 glucose; and gassed with 95% oxygen/5% carbon dioxide to obtain pH 7.4. Physiological bath solution used in the perforated whole cell patch-clamp experiments contained (in mM): 134 NaCl; 6 KCl; 1 MgCl_2_; 2 CaCl_2_; 10 glucose; 10 HEPES; and pH was adjusted to 7.4 with NaOH. The patch-pipette solution had (in mM): 110 potassium aspartate; 30 KCl; 10 NaCl; 1 MgCl_2_; 10 HEPES; 0.05 EGTA; and pH was adjusted to 7.2 with NaOH. Amphotericin-B stock solution was prepared daily in dimethyl sulfoxide (DMSO) and was added to the pipette solution (200-300 µg/ml) before the experiment and replaced as needed. L-364373, retigabine, XE991, and linopiridine were purchased from Tocris Bioscience (Bristol, UK) or Toronto Research Chemicals (Toronto, Ontario, Canada). Stock solutions of L-364373, retigabine, and XE991 were prepared in DMSO. Linopiridine stock solution was made in double-distilled water. All compounds were further diluted in the PSS solution. All other chemicals were purchased from Fisher Scientific Co. (Pittsburgh, PA, USA) or Sigma-Aldrich.

### Data analysis and statistics

We used MyoMed software (Med Associates) to record isometric DSM spontaneous phasic and nerve-evoked contractions. MiniAnalysis software (Synaptosoft, Inc., Decatur, GA, USA) was used for data analysis of DSM spontaneous phasic and nerve-evoked contraction amplitude, muscle force (determined by integrating the area under the curve of the phasic contractions), contraction frequency, phasic contraction duration (determined at the level of amplitude half-width), and muscle tone (determined by the phasic contraction baseline curve).

For DSM spontaneous phasic contractions and 10 Hz EFS-induced contractions, a 5 min period prior to the first concentration addition (100 nM) of L-374373, retigabine, XE991, or linopiridine was taken as a control (100%). The responses to subsequent increasing concentrations of compound applications (up to 30 or 100 µM in half-log unit increments) were normalized to that control. During cumulative compound additions, the effects of each concentration on DSM contraction amplitude, muscle force, frequency, duration, and muscle tone were evaluated by analyzing a 5 min period prior to the subsequent higher concentration application. For the 0.5–50 Hz EFS-induced DSM contractions, the contraction amplitude at EFS frequency of 50 Hz prior to compound application (control condition) was taken to be 100% and the data were normalized. In the perforated whole cell patch-clamp experiments, the average membrane potential values prior to, after the stabilization of the effect, and following the washout of retigabine (10 µM) were determined using the Clampfit ver.10 software (Molecular Devices, Sunnyvale, CA, USA). GraphPad Prism 4.03 software (GraphPad Software, Inc., La Jolla, CA, USA) and Microsoft Excel (Redmond, WA, USA) were used for statistical analyses and CorelDraw Graphic Suite software (Corel Co, Ottawa, Ontario, Canada) was used for data illustration. In the summary data, ***n*** represents the number of individual DSM isolated strips or cells and ***N*** is the number of guinea pigs. Paired Student’s t-test was used to analyze concentration-responses, and frequency-response curves. All results are expressed either as means (95% confidence intervals) for EC_50_/IC_50_ estimates or means±SEM; P<0.05 was considered to be statistically significant.

## Results

### RT-PCR and qRT-PCR experiments demonstrate the expression of Kv7 channel subtypes in guinea pig DSM

**Figure 1:**
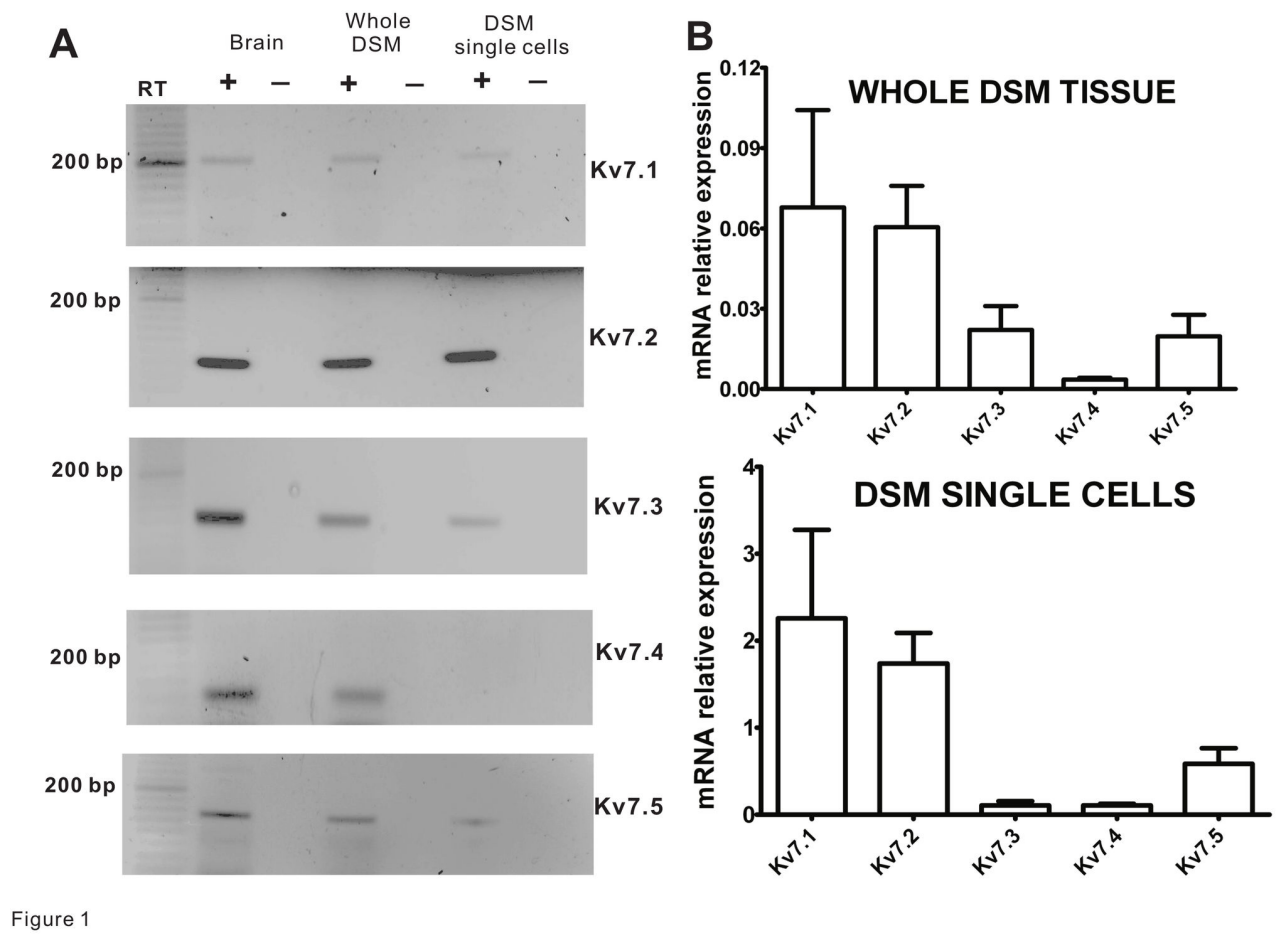
Kv7 channel subtype mRNA expression in guinea pig DSM whole tissue and single-cells. **A**) Gel electrophoresis images illustrate RT-PCR detection of all Kv7 channel subtypes including Kv7.1, Kv7.2, Kv7.3, Kv7.4, and Kv7.5 channel mRNA messages in mucosa-free whole DSM tissue (N=4). At DSM single-cell level, unlike other Kv7 channel subtypes (Kv7.1, Kv7.2, Kv7.3, and Kv7.5), the Kv7.4 channel mRNA message was not detected. Guinea pig brain was used as a positive control. No product was observed in the negative controls (-RT) in which reverse transcriptase was not added to the reaction. **B**) qRT-PCR analysis shows relatively high expression of Kv7.1, Kv7.2, and Kv7.5 subtypes but low (barely detectible) levels of Kv7.4 channel mRNA in both DSM whole tissue (N=4) and DSM single cells (N=4). Data were normalized to GAPDH using the ΔCt method.

We conducted RT-PCR and qRT-PCR experiments with whole DSM tissue and a pool of isolated DSM cells in order to investigate the expression of individual Kv7 channel subtypes at the mRNA level. Consistent with a previous study [38], all Kv7 channel subtypes (transcripts) were detected in guinea pig brain (Figure 1A). In addition, the RT-PCR experiments revealed mRNA expression for all Kv7 channel subtypes in guinea pig whole DSM tissue (N=4; Figure 1A). Since mRNA expression in whole tissue could have originated not just from DSM cells but also from other cell types present in the muscle layer (such as: as neurons, fibroblasts, vascular, and endothelial cells), RT-PCR experiments were carried out on RNA samples collected from isolated DSM cells. Unlike whole DSM tissue, the RT-PCR analysis on DSM single-cells did not show the mRNA expression for the Kv7.4 channel subtype, but it did so for the other four subtypes (N=4; Figure 1A). Negative control experiments performed in the absence of the reverse transcriptase (-RT) demonstrated no bands indicating an absence of genomic DNA contamination (Figure 1A). In order to determine the relative expression of individual Kv7 transcripts in DSM, we conducted qRT-PCR analysis in DSM whole tissue and isolated single-cells. In whole DSM tissue, we found that the relative mRNA Kv7 channel expression levels were as follows: Kv7.1~Kv7.2Kv7.3~Kv7.5Kv7.4 (N=4; Figure 1B). When qRT-PCR analysis was performed on isolated DSM cells, the relative mRNA levels were: Kv7.1~Kv7.2Kv7.5Kv7.3~Kv7.4 (N=4; Figure 1B). These qRT-PCR experiments further suggest that in DSM cells the Kv7.1, Kv7.2, and Kv7.5 channel subtypes are highly expressed at the mRNA level, whereas Kv7.4 channels are not.

### Guinea pig DSM cells express Kv7.1, Kv7.2, Kv7.3, and Kv7.5 channel subtype proteins

**Figure 2:**
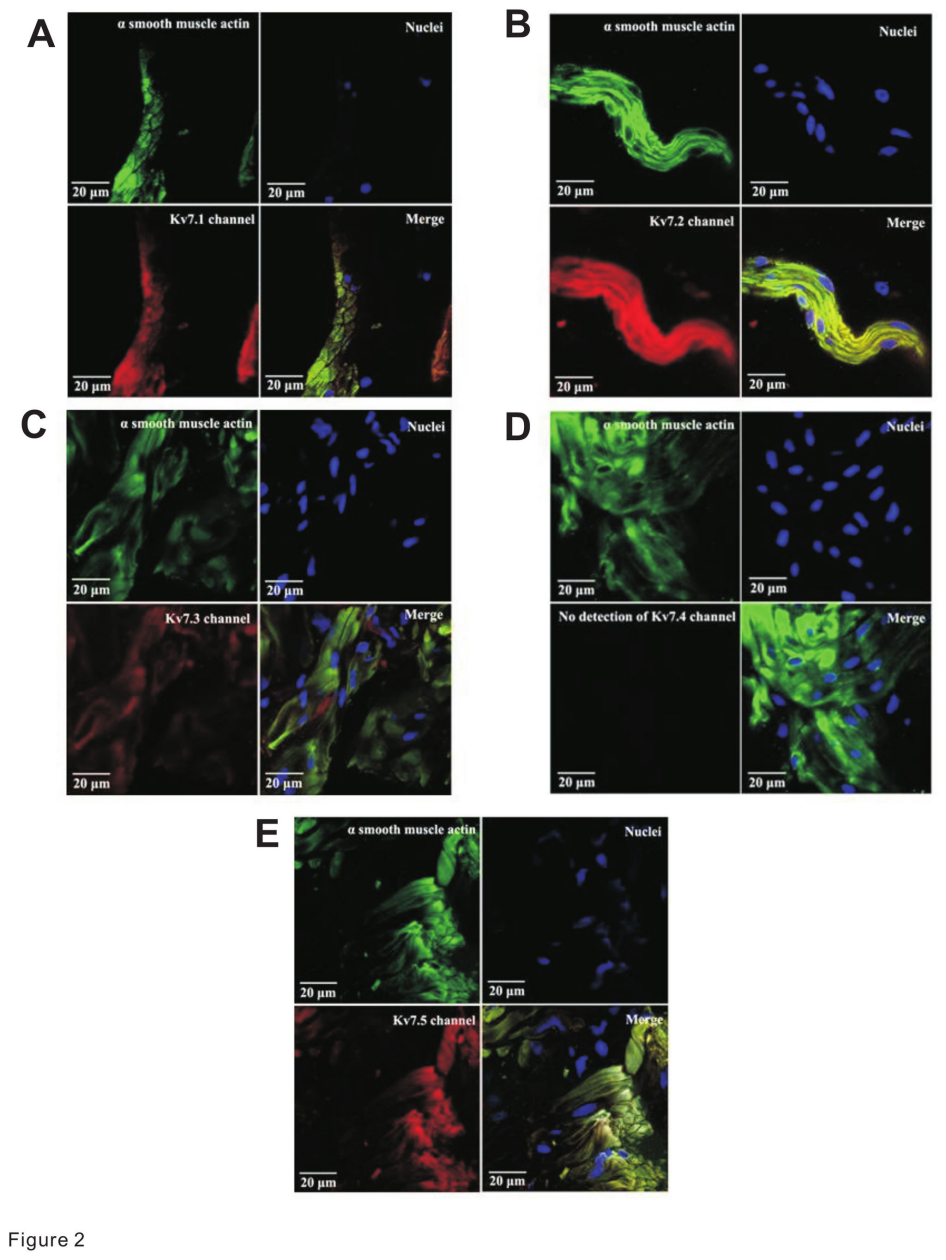
Protein expression of Kv7 channel subtypes in guinea pig DSM. Confocal images illustrate the staining for Kv7.1 (**A**), Kv7.2 (**B**), Kv7.3 (**C**), Kv7.4 (**D**), and Kv7.5 (**E**) channel subtype proteins in mucosa-free whole DSM tissue. Kv7 channel subtype proteins were detected by immunohistochemistry using subtype-specific antibodies. In all panels, α-smooth muscle actin is shown in green; cell nuclei are illustrated in blue; the specific Kv7 channel subtype protein expression is represented by red staining. The merged images of α-smooth muscle actin, nuclei, and the Kv7 channel protein expression are illustrated in the quadrant labeled “Merge”. Images were captured with a Carl Zeiss LSM 700 META confocal microscope (63x objective). Experiments were conducted on DSM tissue samples isolated from 3 different guinea pigs.

To assess whether the Kv7 channel subtypes found at the mRNA level were also expressed at the protein level, we performed immunostaining analyses. Immunohistochemical experiments were done with thin sections of whole DSM tissue co-stained with Kv7 channel subtype-specific antibodies and an α-smooth muscle actin antibody. With the use of confocal microscopy, we were able to determine the specific detection of Kv7 channel subtypes directly in DSM cells identified by co-expression of the two markers within the same regions. We found protein expression for Kv7.1, Kv7.2, Kv7.3, and Kv7.5 channels specifically in DSM cells (Figure 2). However, we did not detect a protein expression for Kv7.4 channels in DSM cells consistent with the single-cell RT-PCR data (N=3; Figure 1A).

### Kv7 channel openers cause inhibition of spontaneous phasic contractions of guinea pig DSM isolated strips

**Figure 3:**
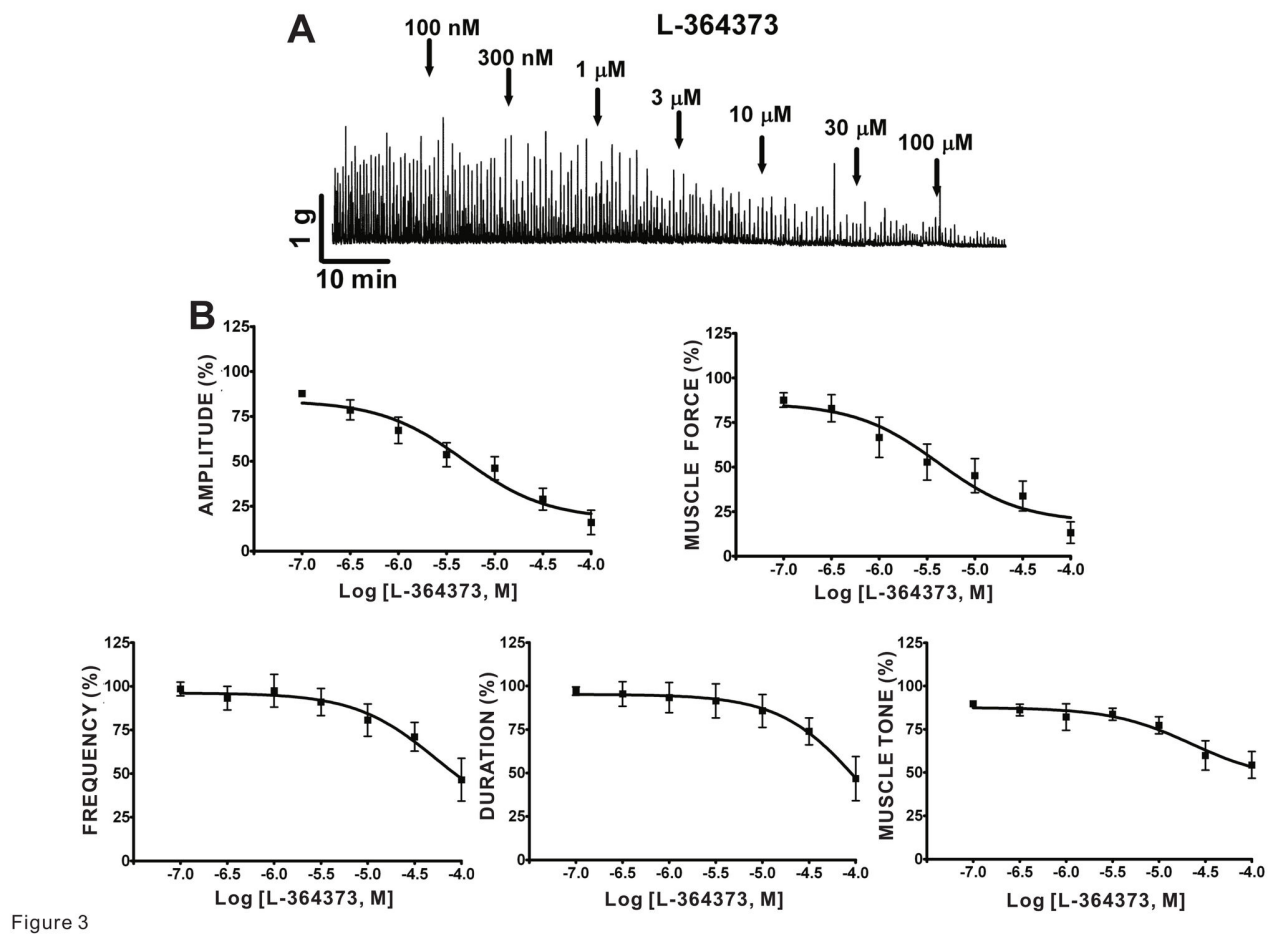
Kv7.1 channel activator L-364373 inhibited spontaneous phasic contractions in guinea pig DSM isolated strips. **A**) This original DSM tension recording illustrates L-364373 inhibitory effect on spontaneous phasic contractions of DSM isolated strips in a concentration-dependent manner. **B**) Cumulative concentration-response curves for L-364373 show significant reduction in DSM spontaneous phasic contraction amplitude, muscle force, frequency, duration, and muscle tone (n=7, N=4); see Table 1 for potency and maximum efficacy values. TTX (1 µM) was present throughout the experiments.

**Figure 4:**
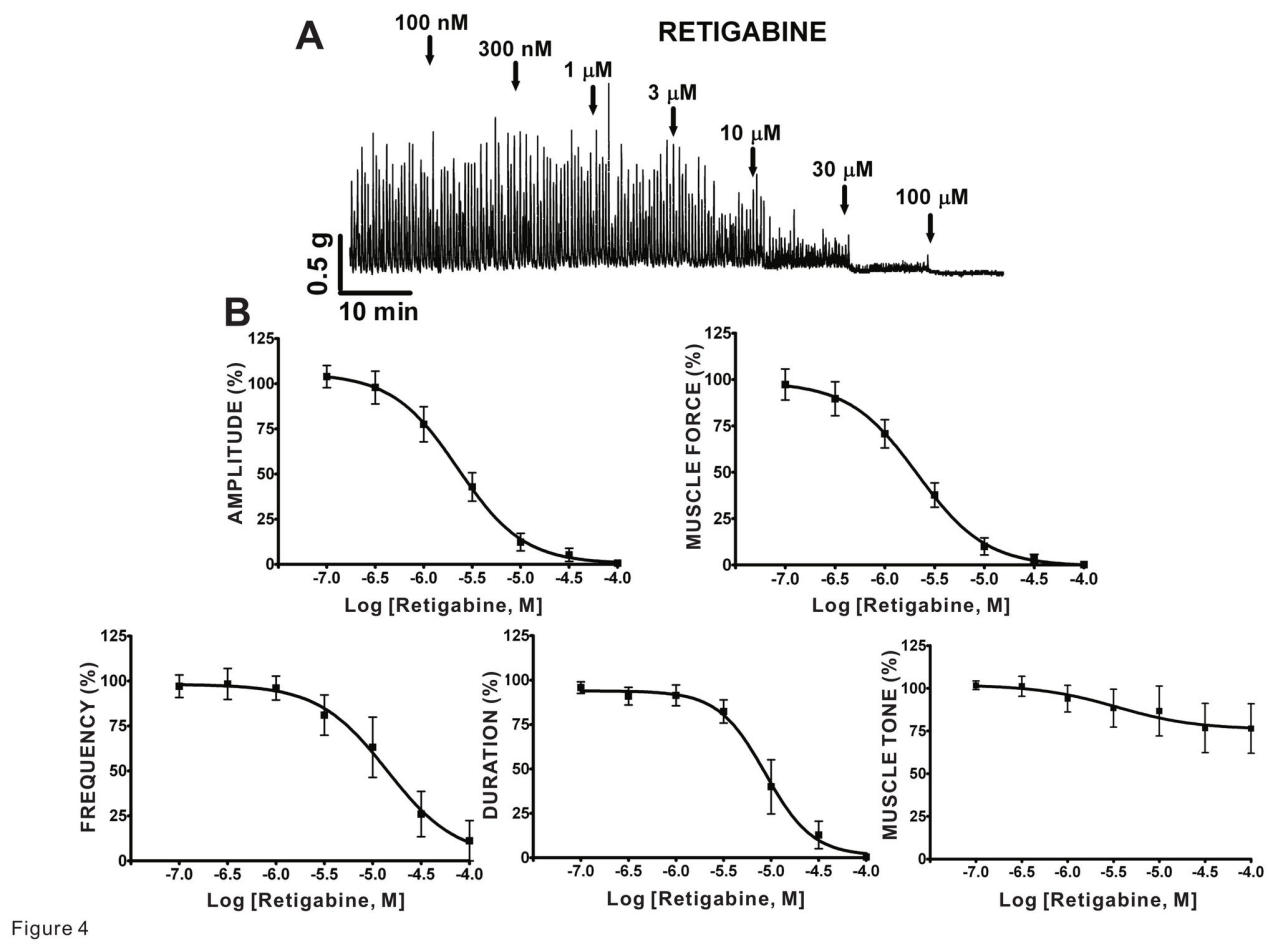
Kv7.2-Kv7.5 channel opener retigabine decreased spontaneous phasic contractions in guinea pig DSM isolated strips. **A**) This original DSM tension recording illustrates retigabine inhibitory effects on spontaneous phasic contractions of DSM isolated strips in a concentration-dependent manner. **B**) Cumulative concentration-response curves for retigabine summarize reduction in DSM spontaneous phasic contraction amplitude, muscle force, frequency, and duration (n=7, N=6); see Table 1 for potency and maximum efficacy values. TTX (1 µM) was present throughout the experiments.

We investigated the effects on DSM spontaneous phasic and tonic contractions to two pharmacologically distinct Kv7 channel activators: L-364373, active at Kv7.1 channels, and retigabine, selective at Kv7.2-7.5 channels, in isolated strips. We found that L-364373 effectively decreased the spontaneous phasic and tonic contractions of DSM isolated strips (Figure 3). In particular, L-364373 attenuated the spontaneous phasic contraction amplitude, muscle force, frequency, duration, and muscle tone of DSM isolated strips in a concentration-dependent manner (n=7, N=4; Figure 3B and Table 1). In comparison to L-364373, retigabine had a more pronounced effect on DSM spontaneous phasic contractions. Retigabine inhibited the spontaneous phasic contraction amplitude, muscle force, frequency, and duration in a concentration-dependent manner (n=7, N=6; Figure 4B and Table 1). These results support that pharmacological Kv7 channel activation leads to inhibition of the spontaneous phasic and tonic contractions in DSM isolated strips.

**Table 1:** Potency and maximum effect values of Kv7 channel modulators on spontaneous phasic and tonic contractions of guinea pig DSM isolated strips.

**Compound**	**Amplitude**	**Muscle Force**	**Frequency**	**Duration**	**Muscle Tone**
**IC_50_/EC_50_: Mean (95% Confidence Interval**)					
**Maximum Efficacy: Mean ± SEM**					
**L-364373**	4.8 (2.0-11.7)µM	4.1 (1.2-13.9)µM	53.5 (6.0-480.4)µM	>100µM	22.4 (4.0-126.9)µM
	16.0±6.8%^###^	13.2±6.0%^###^	46.4±12.2%^##^	46.8±12.7%^##^	54.4±7.7%^##^
**Retigabine**	2.3 (1.4–3.5)µM	2.1 (1.3–3.4)µM	14.0 (4.3–46.1)µM	8.7 (5.6-13.4)µM	3.5 (0.1–17.3)µM
	0.7±0.7%^###^	0.2±0.2%^###^	11.2±11.2%^###^	0.4±0.4%^###^	76.4±14.9%^$^
**XE991**	4.5 (0.4–54.6)µM	2.3 (0.1–49.3)µM	—	—	—
	212.9±42.2%^#^	233.3±66.2%^#^	101.0±11.5%	115.7±15.3%	139.9±54.0%
**Linopiridine**	11.5 (1.0–127.0)µM	10.7 (0.9-132.5)µM	**—**	**—**	**—**
	415.1±75.9%^##^	415.1±110.4%^#^	90.7±15.0%	126.8±21.5%	140.0±11.4%^##^

Maximum effects are reported in comparison to control (taken to be 100%). Each data point is n=7-8, N=4-6; ^#^P<0.05, ^##^P<0.01, ^###^P<0.001, ^$^P=0.07 vs. control.

### Kv7 channel inhibitors enhance spontaneous phasic contractions of guinea pig DSM isolated strips

**Figure 5:**
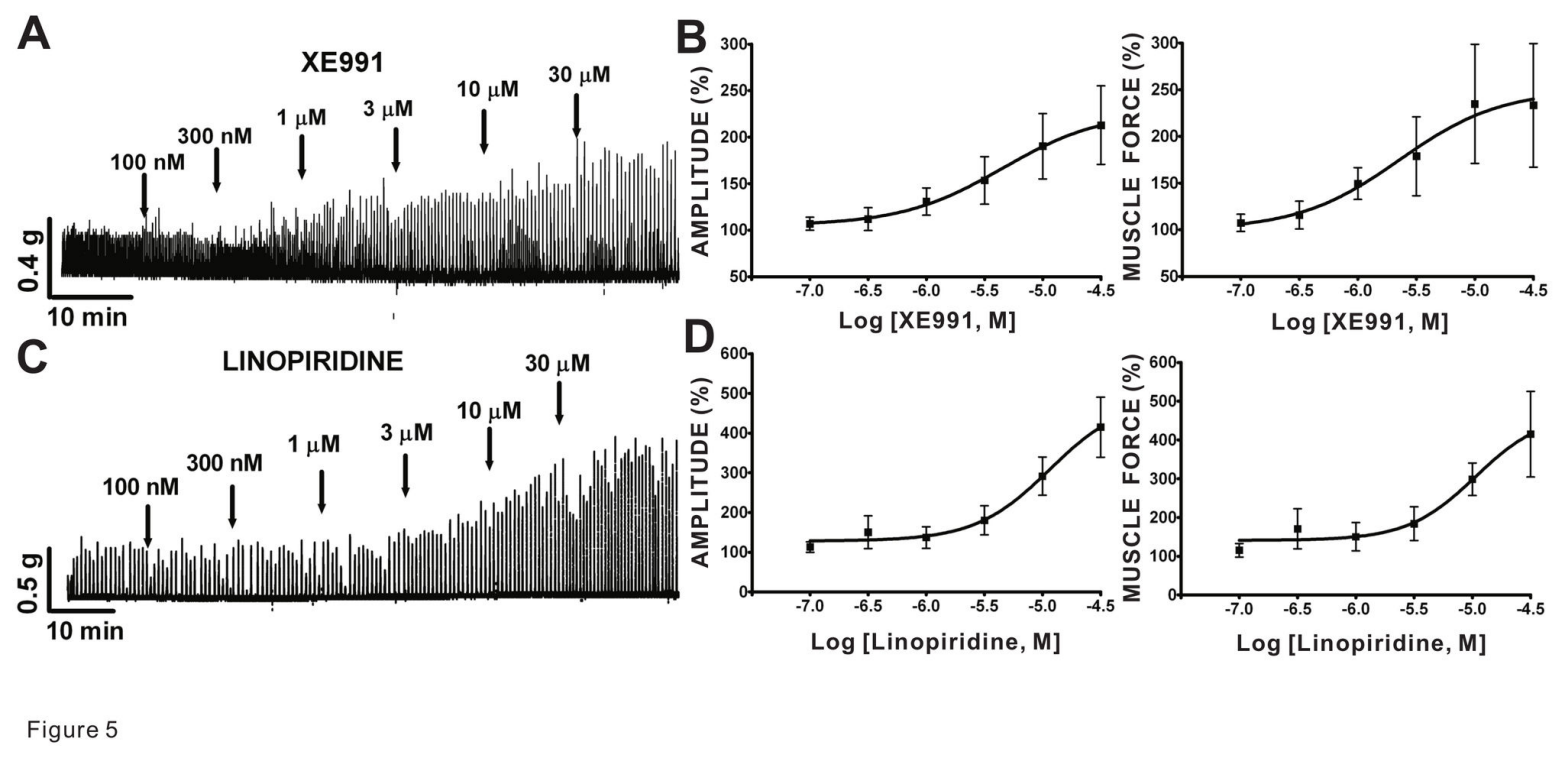
Kv7.1-Kv7.5 inhibitors, XE991 and linopiridine, increased spontaneous phasic contractions in guinea pig DSM isolated strips. **A**) This original DSM tension recording illustrates that XE991 enhances spontaneous phasic contractions in isolated DSM strips in a concentration-dependent manner. **B**) Cumulative concentration-response curves for XE991 show increases in DSM spontaneous phasic contractions amplitude and muscle force (n=8, N=5). **C**) This original DSM tension recording exemplifies that linopiridine increases DSM spontaneous phasic contractions in a concentration-dependent manner. **D**) Cumulative concentration-response curves for linopiridine depict increases in DSM spontaneous phasic contractions amplitude and muscle force (n=7, N=5). Table 1 provides a summary of potency and maximum efficacy values. TTX (1 µM) was present throughout the experiments.

We investigated the effects of pharmacological inhibitors of Kv7 channels on spontaneous phasic and tonic contractions of DSM isolated strips. We used XE991 and linopiridine, two pan-Kv7 channel inhibitors. We found that XE991 significantly enhanced the spontaneous phasic contraction amplitude and muscle force of DSM isolated strips in a concentration-dependent manner without statistically significant effects on contraction frequency, duration, and muscle tone (n=8, N=5; Figure **5A-B** and Table **1**). In comparison, the other Kv7 channel inhibitor linopiridine also increased phasic contraction amplitude and muscle force in a concentration-dependent manner as well as the muscle tone, but this compound did not significantly alter contraction frequency or duration (n=7, N=5; Figure **5C-D** and Table **1**). These data support that Kv7 channel blockade enhances the spontaneous phasic contractions in DSM isolated strips.

### Pharmacological activators of Kv7 channels inhibit EFS-induced contractions of guinea pig DSM isolated strips

**Figure 6:**
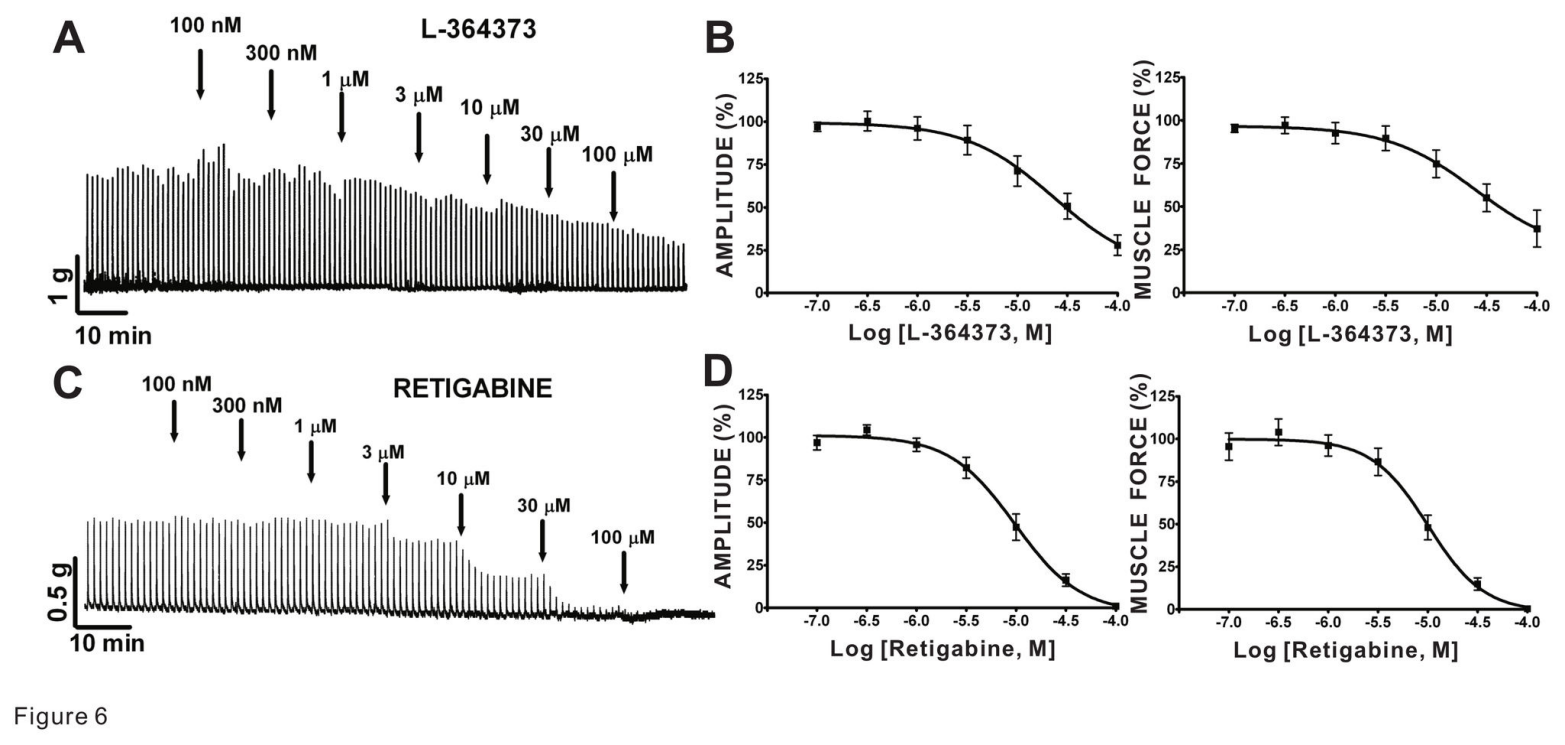
Kv7 channel activators, L-364373 and retigabine, induced inhibition of the 10 Hz EFS-evoked contractions in guinea pig DSM isolated strips. **A**) This original DSM tension recording illustrates L-364373 inhibitory effects on DSM 10 Hz EFS-induced contractions. **B**) Cumulative concentration-response curves for L-364373 summarize inhibitory effects on the amplitude and muscle force of EFS-induced contractions (n=6, N=4). **C**) This original DSM tension recording depicts an effect of retigabine on contractions induced by EFS (10 Hz). **D**) Cumulative concentration-response curves for retigabine demonstrate reduction of 10 Hz EFS-induced DSM contraction amplitude and muscle force (n=9, N=8). Table 2 summarizes the potency and maximum efficacy values.

We investigated the effects of L-364373 and retigabine on the amplitude, muscle force, duration, and muscle tone of EFS-induced DSM contractions generated by 10 Hz/min stimulations in DSM isolated strips. We found that L-364373 attenuated 10 Hz EFS-induced contraction amplitude and muscle force without any significant effects on contraction duration and muscle tone (n=6, N=4; Figure 6B and Table 2). Retigabine also decreased the 10 Hz EFS-induced contraction amplitude and muscle force, and additionally reduced the duration (n=9, N=8; Figure 6D and Table 2). These data substantiate that pharmacological activation of Kv7 channels attenuates nerve-evoked contractions in DSM isolated strips.

**Table 2:** Potency and maximum effect values of Kv7 channel modulators on 10 Hz EFS-induced contractions of guinea pig DSM isolated strips.

**Compound**	**Amplitude**	**Muscle Force**	**Duration**	**Muscle Tone**
**IC_50_/EC_50_: Mean (95% Confidence Interval**)				
**Maximum Efficacy: Mean±SEM**				
**L-364373**	23.1 (8.7–61.2)µM	24.9 (7.3–84.3)µM	—	—
	28.0±6.0%^###^	37.2±10.7%^##^	106.7±8.7%	79.5±14.1%
**Retigabine**	9.7 (6.9–13.9)µM	10.1 (6.7–15.1)µM	40.6 (19.2–86.0)µM	—
	1.0±0.7%^###^	0.3±0.2%^###^	1.1±0.7%^###^	96.7±16.6%
**XE991**	0.6 (0.1–5.6)µM	1.4 (0.2–9.8)µM	—	4.8 (0.3-76.6)µM
	155.3±16.4%^##^	180.5±24.2%^##^	104.8±5.2%	132.9±7.0%^##^
**Linopiridine**	>30µM	6.3 (0.5-79.0)µM	—	>30µM
	144.0±20.1%^#^	130.1±13.1%^#^	94.3±4.0%	159.3±28.6%^#^

Maximum effects are reported in comparison to control (taken to be 100%). Each data point is n=6-9, N=4-8; ^#^P< 0.05, ^##^P< 0.01, ^###^P<0.001 vs control.

### Pharmacological inhibitors of Kv7 channels increase EFS-induced contractions of guinea pig DSM isolated strips

**Figure 7:**
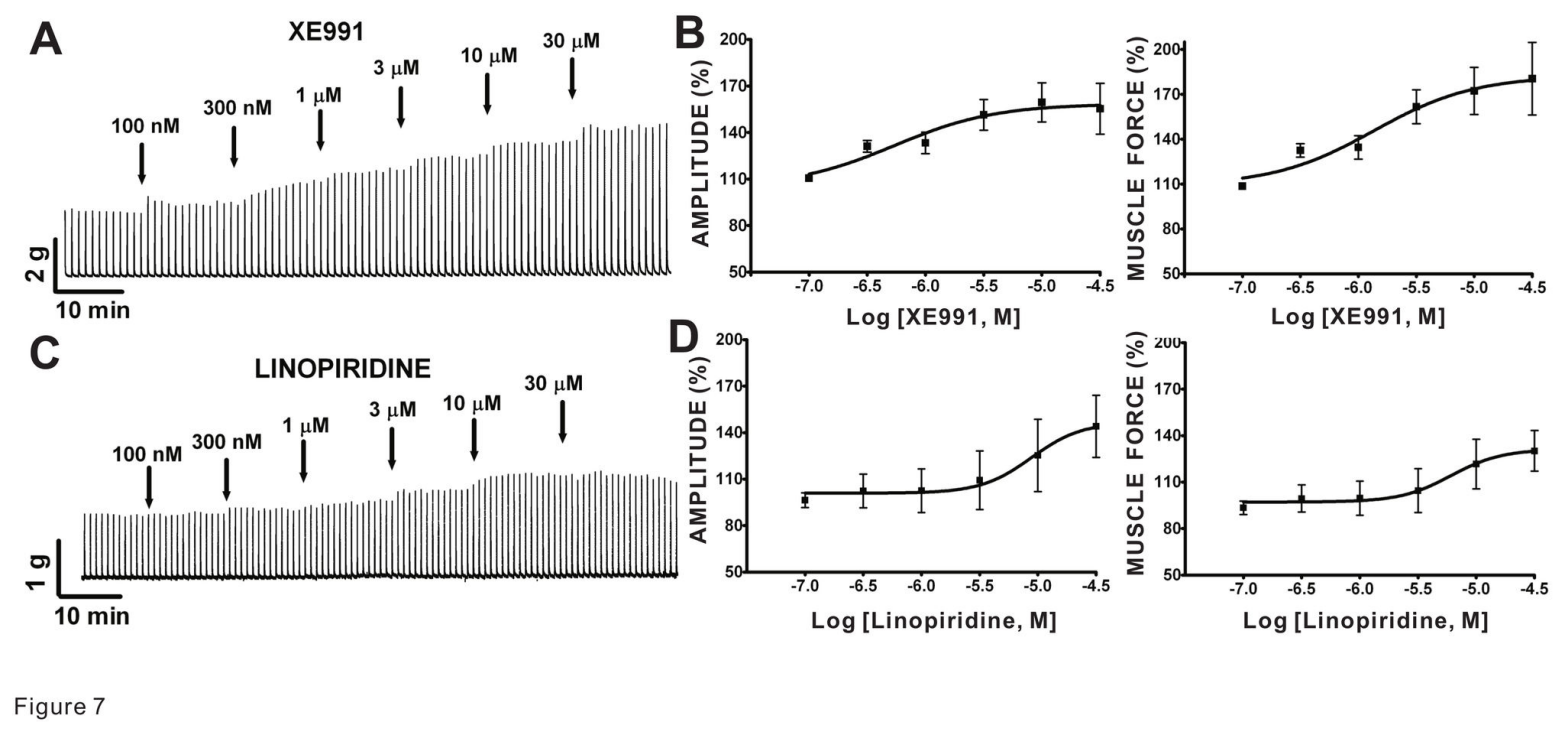
Kv7 inhibitors, XE991 and linopiridine, enhanced the 10 Hz EFS-induced contractions in guinea pig DSM isolated strips. **A**) This original DSM tension recording illustrates that XE991 increased 10 Hz EFS-induced contractions. **B**) Cumulative concentration-response curves for XE991 show potentiation of amplitude and muscle force (n=9, N=7). **C**) This original DSM tension recording for linopridine (100 nM–30 µM) illustrates an increase in 10 Hz EFS-induced contractions. **D**) Cumulative concentration-response curves for linopiridine show inhibition of EFS-induced contraction amplitude and muscle force (n=6, N=5); see Table **2** for the potency and maximum efficacy values.

We used XE991 and linopiridine, two Kv7 channel blockers, to assess the change in 10 Hz EFS-induced contractions in DSM isolated strips upon Kv7 channel blockade. XE991 (n=9, N=7) and linopiridine (n=6, N=5) effectively enhanced the amplitude and muscle force of the 10 Hz EFS-induced contractions of DSM isolated strips in a concentration-dependent manner (Figure 7A-D and Table 2). Both compounds also increased the muscle tone (Table 2). These data support that pharmacological blockade of Kv7 channels enhances nerve-evoked contractions in DSM isolated strips.

### Kv7 channel activators, L-364373 and retigabine, decrease the amplitude of the EFS-induced DSM contractions in a wide range of stimulation frequencies

**Figure 8:**
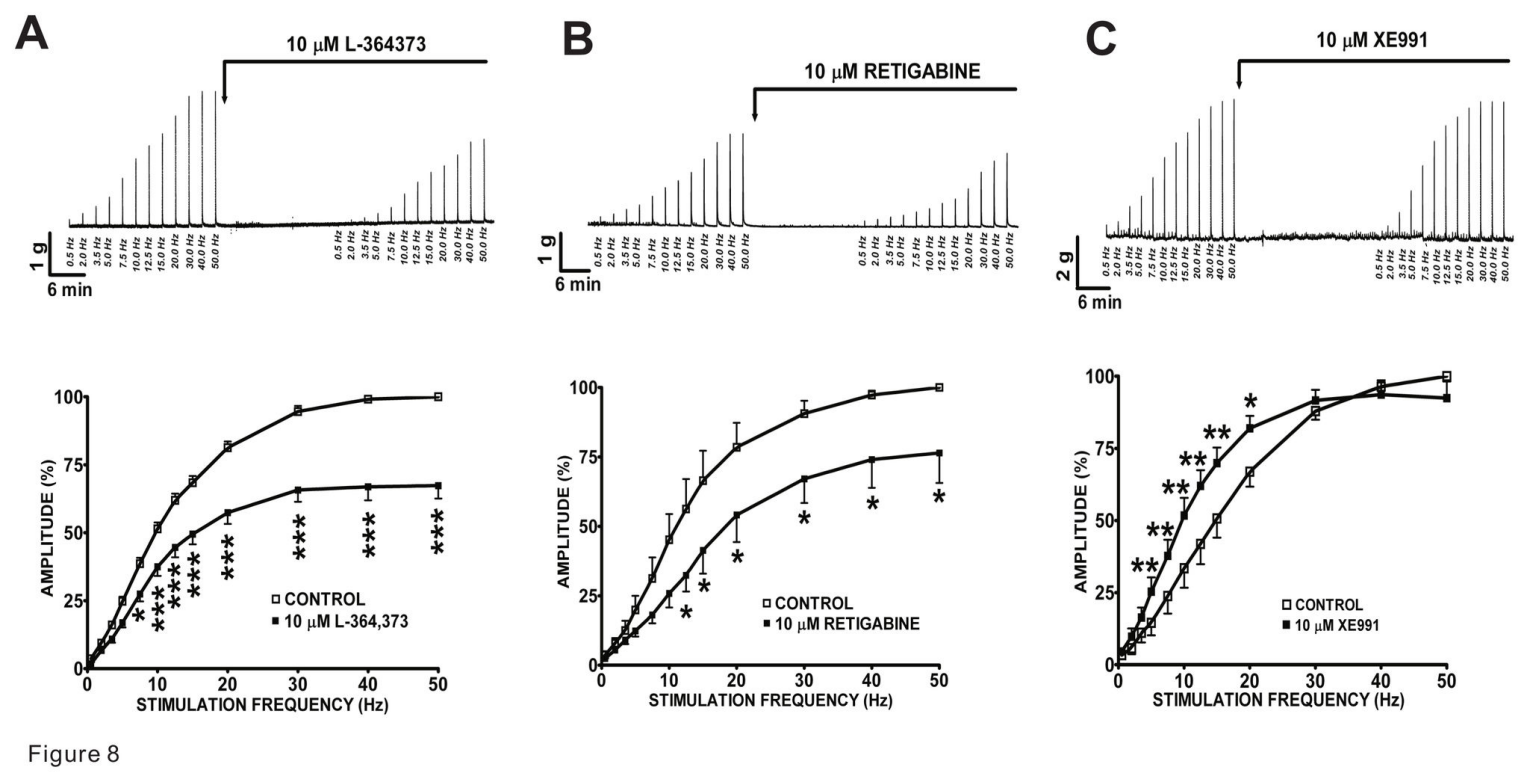
Effect of Kv7 channel modulators on the 0.5–50 Hz EFS-induced contractions in guinea pig DSM isolated strips. **A**-**B**) The original DSM tension recordings and frequency-response curves illustrate the inhibitory effects of L-364373 (n=7, N=4) and retigabine (n=6, N=4) on the 0.5–50 Hz EFS-induced contraction amplitude. **C**) The original DSM tension recording and frequency-response curves reveal that XE991 enhanced the 0.5-20 Hz EFS-induced contraction amplitude (n=8, N=5). The maximal EFS-induced contraction amplitude at a stimulation frequency of 50 Hz under control conditions was taken to be 100%; *P<0.05, **P<0.01, ***P<0.005 versus control.

We addressed how activation of Kv7 channels modulates DSM nerve-evoked contractions in response to a wide range of EFS frequencies in DSM isolated strips. We first applied increasing EFS frequencies (0.5–50 Hz) as a control protocol, followed by the addition of a single concentration of L-364373 (10 µM) or retigabine (10 µM) (Figure 8A-B). Then, a second EFS protocol was applied to evaluate the compound effects on the EFS-induced DSM contractions. We found that in DSM isolated strips, L-364373 (10 µM) inhibited the 7.5–50 Hz EFS-induced contractions (n=7, N=4; Figure 8A). Retigabine (10 µM) also significantly decreased the amplitude of the EFS-induced contractions at stimulation frequencies ranging between 12.5 and 50 Hz (n=6, N=4; Figure 8B). These data indicate that activation of Kv7 channels can reduce nerve-evoked DSM contractions induced by EFS at a wide range of stimulation frequencies.

### Pan-Kv7 channel inhibitor XE991 increases the amplitude of EFS-induced DSM contractions over a wide range of stimulation frequencies

XE991, a Kv7.1-7.5 channel inhibitor, was used to investigate the changes in DSM nerve-evoked contractions. We employed the same experimental approach as described above for the Kv7 channel activators (Figure 8A-B). In DSM isolated strips, XE991 (10 µM) significantly increased the amplitude of the EFS-induced contractions at stimulation frequencies ranging between 5.0 and 20 Hz (n=8, N=5; P<0.001-0.05 for each data point as indicated; Figure 8C). These data support that inhibition of Kv7 channels enhances DSM nerve-evoked contractions, induced by EFS, at a wide range of stimulation frequencies.

### Kv7 channel activator retigabine induces hyperpolarization of freshly-isolated DSM single-cells

**Figure 9:**
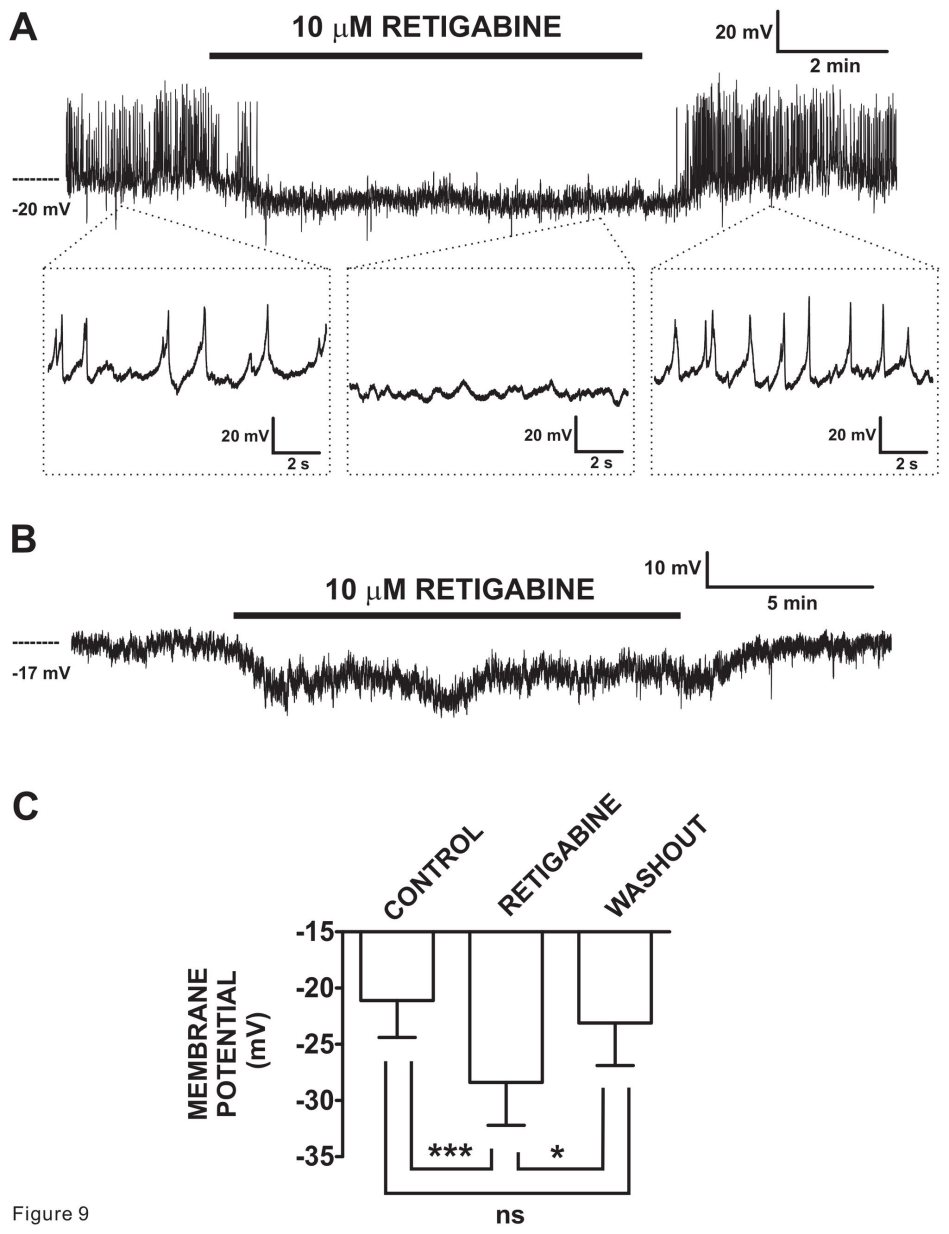
Kv7.2-7.5 channel activator retigabine induced hyperpolarization and inhibition of spontaneous action potentials in freshly-isolated guinea pig DSM cells. **A**) The original current-clamp membrane potential recording illustrates spontaneous action potentials exhibited by a DSM cell. Retigabine (10 µM) inhibited these spontaneous action potentials and caused membrane hyperpolarization. Upon washout, the electrical activity fully recovered. The insets in (**A**) depict the electrical activity on an expanded time scale for the time points indicated allowing for visualization of the action potentials. **B**) The original membrane potential recording from a DSM cell lacking spontaneous action potentials. Retigabine (10 µM) induced hyperpolarization and upon its washout membrane potential recovered. **C**) Summary data show statistically significant hyperpolarization of DSM cells by retigabine (10 µM) (n=12, N=11) and the recovery upon its washout (n=8, N=7). The bars depict actual mean membrane potential and SEM values for each condition. The indicated comparisons indicate statistical significance of ***P<0.001 and *<0.05 for the specified conditions, ns = non-significant (P>0.05).

To assess the effect of retigabine on DSM cell excitability, membrane potential recordings were made in 12 cells (N=11), with cell capacitance of 39.8 ± 2.9 pF using the perforated whole cell patch-clamp technique, which preserves the intracellular native signaling environment. Under control conditions, the DSM cells exhibited variable types of electrical activities as reported previously [28,33,34,36,39]. Spontaneous action potentials were recorded in 33% of all DSM cells studied (Figure 9A). The addition of retigabine (10 µM) caused inhibition of the action potentials associated with membrane hyperpolarization. In the remaining cells without spontaneous action potentials, exemplified by Figure 9B, retigabine also induced hyperpolarization. Collectively, the average membrane potential values were -21.1 ± 3.3 mV and -28.4 ± 3.8 mV (P<0.001, n=12, N=11; Figure 9C) in the absence and presence of retigabine (10 µM), respectively. In the majority of the cells tested, we also evaluated the washout of retigabine. As shown in Figure 9A and 9B, there was recovery in both the generation of spontaneous action potentials and the average membrane potential. Following the washout, the membrane potential value returned to -23.1 ± 3.8 mV (n=8, N=7; P>0.05 versus control; Figure 9C). These experiments indicate that the Kv7.2-7.5 channel activator, retigabine, can regulate DSM cell excitability by inducing hyperpolarization and inhibiting spontaneous action potentials, which drive phasic contractions.

## Discussion

The present study reveals the expression at both mRNA and protein levels for Kv7.1, Kv7.2, Kv7.3, and Kv7.5 channel subtypes in guinea pig DSM cells. Additionally, we provide evidence for their critical role in regulating DSM function *in vitro*. We report attenuation of DSM spontaneous and EFS-induced contractions by L-364373 and retigabine, two Kv7 channel activators with distinct pharmacological profiles. We also identified increases in DSM spontaneous and EFS-induced contractions by XE991 and linopiridine, both pan-Kv7 channel blockers, as well as membrane hyperpolarization and inhibition of spontaneous action potentials by retigabine in freshly-isolated DSM cells. The absence or minimal expression of Kv7.4 channels in DSM cells further suggests that the observed changes in DSM contractility by the tested Kv7 channel modulators were mediated via other Kv7 channel subtypes. The native Kv7 channel assemblies most likely involve the homomeric Kv7.1 channel subtype, and homomeric or heteromeric channel complexes containing Kv7.2, Kv7.3, and/or Kv7.5 subtypes.

Our group and others have previously identified the expression of multiple members of the Kv channel family in DSM - including Kv1.2, Kv1.3, Kv1.5, Kv1.6, Kv2.1, Kv5.1, Kv6.1, Kv6.2, Kv6.3, and Kv9.3 channels - regulating various aspects of DSM excitability and contractility [34,40,41,42,43,44]. In this study, RT-PCR experiments performed on enzymatically-isolated guinea pig DSM single-cells demonstrated mRNA expression of Kv7.1, Kv7.2, Kv7.3, and Kv7.5 channel subtypes but not the Kv7.4 channel. In contrast, in whole DSM tissue mRNA messages for all Kv7 channel subtypes were detected. Further qRT-PCR experiments showed the following rank order of transcript expression in isolated DSM cells: Kv7.1~Kv7.2Kv7.5Kv7.3~Kv7.4 and a similar profile in whole DSM tissue except for the higher expression of Kv7.4 channels. This suggests that the primary source of Kv7.4 mRNA originates from non-DSM cells. To detect protein expression of Kv7 channel subtypes in DSM, immunohistochemical analyses were conducted using specific antibodies for each Kv7 channel subtype, α-smooth muscle actin antibody, and confocal microscopy allowing for determination of co-localization of the two markers. As illustrated in Figure 2, we could not detect any specific staining for Kv7.4 in DSM cells but did so for Kv7.1, Kv7.2, Kv7.3, and Kv7.5 channel subtypes. Our findings further extend and complement the recent observations in guinea pig, pig, and rat DSM [23,25,28]. In guinea pigs, all five Kv7 channel subtypes were detected by RT-PCR and immunohistochemistry [28] confirming our findings for Kv7.1, Kv7.2, Kv7.3, and Kv7.5 channel subtypes. The discrepancy for the Kv7.4 subtypes expression may be due the experimental conditions including the use of different antibodies (both primary and secondary). Furthermore, Anderson et al. [28], unlike our confocal microscopy data presented herein (Figure 2), did not detect the expression of Kv7 channel subtypes by co-labelling with the α-smooth muscle actin marker [28]. Thus, our approach allowed us to directly identify localization within DSM cells. In contrast, in the study by Anderson et al. [28], it was unclear if the detection of Kv7.4 channels originated from DSM cells or another cell type. Using RT-PCR, we detected a relatively weak signal for the Kv7.4 channel subtype in whole tissue but not at all in DSM single cells consistent with the expression of this protein in a non-DSM cell type. By comparison in rat DSM, Svalo et al. [23] found Kv7.4 mRNA and protein expression (although the band in the Western blot was very faint) - but not for Kv7.2 channel protein in DSM whole tissue. Unlike our report, Svalo et al. [23] did not investigate the expression of Kv7.4 nor any other Kv7 channel subtype in DSM cells. Thus, the expression of Kv7.4 could have originated from non-DSM cells found in the DSM whole tissue. In rat DSM whole tissue, RT-PCR or qRT-PCR analyses in addition to Kv7.4 also detected Kv7.5 mRNA expression and either failed to identify the expression of Kv7.1, Kv7.2 and Kv7.3 channel subtypes [27] or showed the expression of the Kv7.1 channel and barely detectible presence, if any, of Kv7.2 and Kv7.3 channel transcripts [23]. Further, a prior patent publication revealed the highest transcript expression of Kv7.5 similar to Kv7.1 followed by Kv7.3 and non-detectible levels of Kv7.2 and Kv7.4 channels in rat DSM [45]. Hence, the expression profile of Kv7 channel subtypes in rat bladder, especially in DSM cells, remains to be clarified and could be different from that in guinea pig or human DSM. In addition, in murine bladder, a robust Kv7.5 mRNA expression was detected [46], and transcripts for Kv7.5 along with Kv7.3 and Kv7.4 channel subtypes were detected in pig urinary bladder [25]. Taken together, our findings and those of Anderson et al. [28] in guinea pigs, and the previous studies in rats [23,45], pigs [25], and humans [45], support that Kv7.5–containing channels could be important regulators of DSM function across different species.

Kv7.5 channels form homometric tetramers or co-assemble with Kv7.3 subunits as Kv7.3/Kv7.5 heteromers [47]. Both combinations could be potentially expressed in DSM cells. Kv7.2 channel subunits can co-assemble with Kv7.3 subunits and also express as independent homomers [47,48]. Since we observed a higher expression of Kv7.3 than Kv7.2 channels both in DSM whole tissue and single-cells (Figure 1B), it is likely that Kv7.2/Kv7.3 heteromers do not constitute a primary native Kv7 channel assembly in DSM cells. Furthermore, as Kv7.1 channels do not assemble with any Kv7.2-Kv7.5 subtypes [49], their expression points to yet another native Kv7 channel type expressed. Taken together, multiple Kv7 channel subunit assemblies are likely expressed in DSM cells and potentially regulate distinct aspects of DSM function.

At present, several Kv7 channel modulators are available for studying the functional roles of these channels. The most notable retigabine, the first-in-class clinically approved Kv7 channel opener [50], potentiates the activities of the Kv7.2–Kv7.5 channels with EC_50_ values in the range of 1-10 µM [11]. In contrast, L-364373 is a Kv7.1 channel activator, which relaxes arteries [51,52]. Both compounds inhibited guinea pig DSM spontaneous contractions supporting a role of at least two distinct types of Kv7 channel assemblies. A similar level of relaxation was observed in rat DSM with retigabine [23,24] and in guinea pig DSM with two other Kv7 channel activators, flupirtine and meclofenamic acid [28]. In addition, we studied the effects of XE991 and linopiridine, two pan-Kv7 channel inhibitors that do not selectively distinguish among the different subtypes [11]. Linopiridine provides an advantage over XE991 as the latter compound, unlike the former, may also inhibit Kv1.2/Kv1.5 or Kv2.1/Kv9.3 channels [3]. Both inhibitors increased spontaneous DSM contractility in guinea pig DSM (Figure 5) similar to a previous report [28]. Our study is the first to characterize the effects of linopiridine on DSM contractility using the full concentration-responses determining the potency and maximum efficacy values extending the previous pharmacological characterization by Anderson et al. [28] conducted using only a single concentration tested. XE991 has been previously shown to increase rat DSM contractility [23,24], similar to those observed in pig and guinea pig DSM (this study) [25,28].

The findings of DSM relaxation by Kv7 channel activation *in vitro* provide the underlying mechanism for the demonstrated efficacy of retigabine *in vivo* [21,22,23] by identifying the DSM cells as a site for pharmacological action. In support, intravesical application of retigabine increased micturition volume and voiding intervals in freely moving conscious rats [22]. Further, in rats with capsaicin-induced irritated bladders, retigabine decreased bladder pressure and delayed voiding *in vivo*, and reduced bladder rings contractility *in vitro* [23]. Kv7 channel subtypes, likely present in both DSM cells and interstitial cells, play a role in the regulation of DSM function *in vitro* and *in vivo*. Our molecular and immunohistochemical data identified the presence of Kv7 channel subtypes directly in DSM cells, while functional evidence supports the existence of Kv7 channels in interstitial cells [26] and in DSM cells (present study) [28]. Importantly, Kv7 channels may regulate the generation of Ca^2+^ oscillations controlling excitability and contractility in DSM cells [28].

In the urinary bladder, Kv7 channel subtypes could also be present in bladder nerves. As such, they may regulate nerve-evoked DSM contractions. We found for the first time that the two different Kv7 channel activators, L-364373 and retigabine, decreased the amplitude and muscle force of DSM EFS-induced contractions in a concentration-dependent manner in guinea pigs (Figure 6). In contrast, both inhibitors of Kv7 channels, XE991 or linopiridine, enhanced the nerve-evoked DSM contractions (Figure 7). Our data are consistent with the effects of retigabine and XE991 identified in rat and pig DSM for nerve-evoked contractions [24,25]. These data clearly support that Kv7 channels play an important regulatory role in guinea pig DSM nerve-evoked contractions.

A comparison of the relaxant effects of retigabine and L-364373 on spontaneous phasic and EFS-induced DSM contractions (amplitude and muscle force, Table 1 versus Table 2) revealed ~4-6-fold higher sensitivity for the myogenic-mediated contractility. This may be due to the known regulation of Kv7 channel activity by pathways induced downstream of muscarinic receptor activation. In fact, Kv7 channels have been originally named acetylcholine-sensitive muscarinic K^+^ channels or “M-type” K^+^ channels [53]. A net result of activation of muscarinic-dependent pathways - including breakdown of phosphatidylinositol 4,5-bisphosphate (PIP_2_), and increases in activities of protein kinase C and Ca^2+^/calmodulin [14] - is inhibition of Kv7 channel activity. Thus, under EFS-induced release of ACh and ATP [8], the activation of muscarinic receptors is expected to attenuate Kv7 channel activity and work to oppose the stimulatory effects of retigabine or L-364373 on Kv7 channels.

The observed molecular expression of Kv7 channel subtypes in DSM cells and the effects of Kv7 channel modulators on DSM contractility add to the growing awareness of the importance of Kv7 channels in controlling smooth muscle function. As such, Kv7 channel subtypes have been also found in the myometrium [4], stomach [16,17], intestine [17], vascular [5,7,52,54,55,56], and airway smooth muscle [2]. Interestingly, the relative expression of the different Kv7 subtypes appears organ/tissue-specific. For example, in the heart, aorta, and brain of rats the relative transcript expressions were Kv7.1Kv7.4Kv7.2Kv7.3~Kv7.5, Kv7.1~Kv7.4Kv7.5Kv7.3~Kv7.1, and Kv7.3Kv7.2~Kv7.5Kv7.4Kv7.1, respectively [23]. In the pig, differential expression of Kv7.3, Kv7.4, and Kv7.5 was found in the brain, heart, and urinary bladder [25]. In comparison, in guinea pig DSM cells we observed the following expression profile: Kv7.1~Kv7.2Kv7.5Kv7.3~Kv7.4 and in whole DSM tissue: Kv7.1~Kv7.2Kv7.3~Kv7.5Kv7.4 (Figure **1B**). Generally in non-bladder organs, Kv7 channel activators, such as retigabine and flupirtine, cause relaxation of isolated smooth muscle strips; Kv7 channel inhibitors have an opposite effect [11,13]. We and Anderson et al. [28] noted similar effects in guinea pig DSM strips. Using the perforated whole cell patch-clamp technique, which preserves the native cell environment, retigabine induced membrane hyperpolarization and inhibited spontaneous action potential in freshly-isolated DSM cells (Figure **9**). The effects were fully reversible. To our knowledge, our report is the first to show the hyperpolarizing effect of retigabine and to record spontaneous action potentials with the perforated patch-clamp technique in DSM cells. Previously, spontaneous action potentials were recorded using the conventional whole cell method [28,39], which unlike the perforated patch-clamp technique removes intracellular environment and is, thus, not ideal. The spontaneous action potentials recorded in freshly-isolated guinea pig DSM cells (Figure 9A) resemble the electrical activity measured in DSM tissue strips with microelectrodes [29,57,58]. However, the membrane potential values presented herein (Figure 9), comparable to the previous reports using the same experimental recording conditions (perforated whole cell patch-clamp) [33,34,36,43], revealed more depolarized cell membrane potentials than measured in intact tissues with intracellular microelectrodes [29,57,58]. This reflects the property of isolated DSM cells following enzymatic dissociation measured at room temperature. One of the ion channels likely contributing a depolarizing influence in DSM cells is the Ca^2+^-activated monovalent cation permeable transient receptor potential melastatin 4 (TRPM4) channel [35]. Our results with retigabine complement previous findings measured with the standard whole cell technique demonstrating hyperpolarization or increase in outward currents in DSM cells by Kv7 channel activators, flupirtine and meclofenamic acid, and the opposite effects observed with Kv7 channel inhibitors: XE991, linopirtine and/or chromanol 293B [28]. Furthermore, Kv7 channel currents have been recorded in native myocytes isolated from vascular [6,7,15,54,59,60] and airway smooth muscle [2], as well as in bladder interstitial cells [26]. Similarly, in these smooth muscle cell types under current-clamp recording conditions, Kv7 channel openers (e.g. retigabine) and inhibitors (e.g. XE991) induce hyperpolarization and depolarization, respectively [6,7,26,60]. Thus, Kv7 channel subtypes expressed in DSM and other smooth muscle cell types likely provide fundamental regulation of cell excitability whose roles and functions still remain to be fully characterized and appreciated, especially in the DSM.

## Conclusions

Our study reveals that Kv7 channels are important regulators of guinea pig DSM function as demonstrated by their ability to control spontaneous phasic and EFS-induced DSM contractions and to regulate the membrane potential and spontaneous action potential generation. The changes in guinea pig DSM contractility, observed after application of various Kv7 channel modulators, are mediated by at least two types of Kv7 channels incorporating Kv7.1 and another type consisting of Kv7.2, Kv7.3, and/or Kv7.5 channel subtypes. Our results do not support a major role of Kv7.4 channels in DSM cells. The identification of Kv7 channels in DSM cells and their functional role in regulating DSM contractility provides evidence for their potential utility as promising novel targets for the treatment of urinary bladder dysfunction.
